# Editorial: Antimicrobial resistance dissemination and horizontal gene transfer

**DOI:** 10.3389/fcimb.2023.1240680

**Published:** 2023-07-06

**Authors:** Mingyu Wang

**Affiliations:** State Key Laboratory of Microbial Technology, Microbial Technology Institute, Shandong University, Qingdao, China

**Keywords:** antimicrobial resistance, horizontal gene transfer, one health, agriculture, research topic, editorial

Resistance to antibiotics has raised global awareness for its potential damage to human health, as well as how fast it develops. Not only does antibiotic resistance commonly appear in merely a few years after clinical application, it is also found in nearly every corner of the biosphere soon after emergence, resulting in worldwide consequences. Therefore, the dissemination of antibiotic resistance, *via* mechanisms such as horizontal gene transfer, amplifies the impact of antibiotic resistance mechanisms, and is a leading cause for the severity of the antibiotic resistance problem.

The Research Topic “*Antimicrobial resistance dissemination and horizontal gene transfer*”, published in Frontiers in Cellular and Infection Microbiology, focuses on the dissemination of antimicrobial resistance, and provides in-depth discussions on its prevalence worldwide. Tartor et al. investigated the presence of polymyxin resistance-conferring *mcr* genes in isolated Gram-negative strains from bovine milk in Egypt, and found that the presence of transferrable *mcr* genes is the primary reason for colistin resistance. Igbinosa et al. focused on Methicillin-Resistant *Staphylococcus aureus* (MRSA) from retail poultry meat in Nigeria, and isolated 110 strains from 368 poultry meat samples. Eighty-nine of the 110 strains were found to be multidrug resistant strains, including three that are resistant to all tested antibiotics. Analysis of virulence factors and biofilm formation potentials further demonstrated threats from these bacteria. Liu et al. took a genetics-centered approach, and studied the carriage of β-lactamase genes and *mcr* genes in multidrug *Escherichia coli* isolates from animals in China, and suggested significant differences between isolates from different animals, although similar prevalence of ESBL-producing *E. coli* was observed. A new mutation in *bla*_NDM-5_-carrying plasmid was also discovered. Finally, the mobile colistin resistance mechanisms in non-fermentative Gram-negative bacilli were reviewed by Khuntayaporn et al., summarizing what we know about transmissible resistance to polymyxin, a last-resort antibiotic that has been subject to extensive studies in recent years.

The research papers published in this Research Topic uniformly analyzed the prevalence and dissemination of antibiotic resistance in animals and animal products. This reflects a common concern that the agricultural sector can pose a threat from the perspective of antibiotic resistance. The emergence of mobile polymyxin resistance (*mcr* genes, Liu et al., 2016), as studied and reviewed in articles from this Research Topic, has been an excellent example on how indiscriminate applications of antibiotics in agriculture can lead to serious clinical consequences. Additionally, the lack of application of aseptic techniques in the meat and milk industry, as shown in the prevalence of colistin-resistant bacteria in unpasteurized milk (Tartor et al.), can lead to increased dissemination of the antibiotic resistance.

Research reported in this Research Topic again reminds us to embrace the “One Health” agenda, as the agriculture, the environment, and the clinics are all connected in the only biosphere within the sole planet that all known living systems share. We cannot expect to harm one sector without resulting in harsh consequences in another. With what we have reported in this Research Topic, we hope that measured application of antibiotics can be advocated and promoted in all activities, with special focus on the agricultural sector, so that our shared fragile ecosystem on planet Earth can be well protected, and collateral damage associated with human health can be avoided.

## Author contributions

The author confirms being the sole contributor of this work and has approved it for publication.
